# A penetrating atherosclerotic ulcer rupture in the ascending aorta with hemopericardium: a case report

**DOI:** 10.1186/s13019-016-0502-3

**Published:** 2016-07-11

**Authors:** Yuan-Hao Liu, Hung-Yen Ke, Yi-Chang Lin, Chien-Sung Tsai

**Affiliations:** Division of Cardiovascular Surgery, Department of Surgery, Kaohsiung Armed Forces General Hospital, Kaohsiung, Taiwan, Republic of China; Division of Cardiovascular Surgery, Department of Surgery, Tri-Service General Hospital, National Defense Medical Center, Taipei, Taiwan, Republic of China; Division of Cardiovascular Surgery, Department of Surgery, Taoyuan Armed Forces General Hospital, Taoyuan, Taiwan, Republic of China; Department of Biological Science and Technology, National Chiao Tung University, Hsinchu, Taiwan, Republic of China

**Keywords:** Acute aortic syndrome, Penetrating atherosclerotic ulcer, Ascending aorta, Endovascular repair, Case report

## Abstract

**Background:**

Acute aortic syndrome, including classic aortic dissection, intramural aortic hematoma, and penetrating atherosclerotic ulcer (PAU), is a term used to describe a group of conditions with similar clinical symptoms, but with different pathophysiological mechanisms. PAU is a lesion that penetrates the internal elastic lamina through the media. It is usually located in the descending aorta and rarely observed in the ascending aorta.

**Case presentation:**

A 76-year-old man with a history of essential hypertension was brought to the emergency department (ED) because of a sudden-onset chest pain at rest. He had not been taking his medication as ordered. His vital signs in the ED were a blood pressure of 82/60 mmHg, heart rate of 158 beats per min, respiratory rate of 22 breaths per min, and a body temperature of 37.2 °C. An electrocardiogram did not show an ST segment elevation, and cardiac enzymes were within normal limits. No widening mediastinum was found on chest radiography, but a large pericardial effusion with an impending cardiac tamponade was revealed on echocardiography. The diagnosis of PAU rupture in the ascending aorta with hemopericardium was made with chest computed tomography. An emergent sternotomy and ascending aorta reconstruction were performed. A ruptured ulcerative plaque through the intima to the adventitia without flap dissection in the ascending aorta was confirmed. The patient was discharged 18 days after the operation.

**Conclusions:**

Although PAU in the ascending aorta is uncommon, it is commonly lethal when it ruptures. With the current advances in endovascular techniques and devices, endovascular repair of PAU in the ascending aorta is currently recommended only for high-risk patients unsuitable for open repair. However, we anticipate that endovascular repair may become feasible in patients with PAU in the ascending aorta in the future.

## Background

Acute aortic syndrome (AAS), including classic aortic dissection, intramural aortic hematoma, and penetrating atherosclerotic ulcer (PAU), is a term used to describe a group of conditions with similar clinical symptoms, but with different pathophysiological mechanisms. PAU was first described by Shennan et al. [1] in 1934, but was first shown to be a separate clinical and pathological entity by Stanson et al. [2] in 1986. It is an ulcerating atherosclerotic lesion that disrupts the internal elastic lamina and is associated with hematoma formation within the aortic wall. In the initial stage, atheromatous ulcers develop in patients with advanced atherosclerosis; lesions are usually asymptomatic and confined to the intimal layer. In the second stage, the lesion progresses to a deep atheromatous ulcer that penetrates through the elastic lamina into the media. Hematoma formation may extend along the media, resulting in either a “double-barreled” or “thrombosed” aortic dissection. In some cases, hematoma extension causes stretching of the weakened aortic wall, leading to the formation of a saccular aortic aneurysm [3]. Generally, patients become PAU symptomatic in the second stage, with the main symptom being severe, acute chest pain that radiates to the inter-scapular area; this is initially difficult to differentiate from a classic acute aortic dissection or intramural aortic hematoma. A chest spiral computed tomography (CT) scan provides a short examination time and high quality two- and three-dimensional image reconstruction to help us identify the type of AAS. Occasionally, some patients exhibit several or all of these lesions, indicative of the existence of a link between them. In such cases, it is difficult to know which was the initiating event [4].

Here we present a rare case of AAS in which echocardiography revealed hemopericardium and cardiac tamponade without obvious false lumen in the ascending aorta. Chest CT showed a ruptured PAU in the ascending aorta. An emergent sternotomy and ascending aorta reconstruction were immediately performed. The clinical presentation and management is discussed below.

## Case presentation

**Fig. 1:**
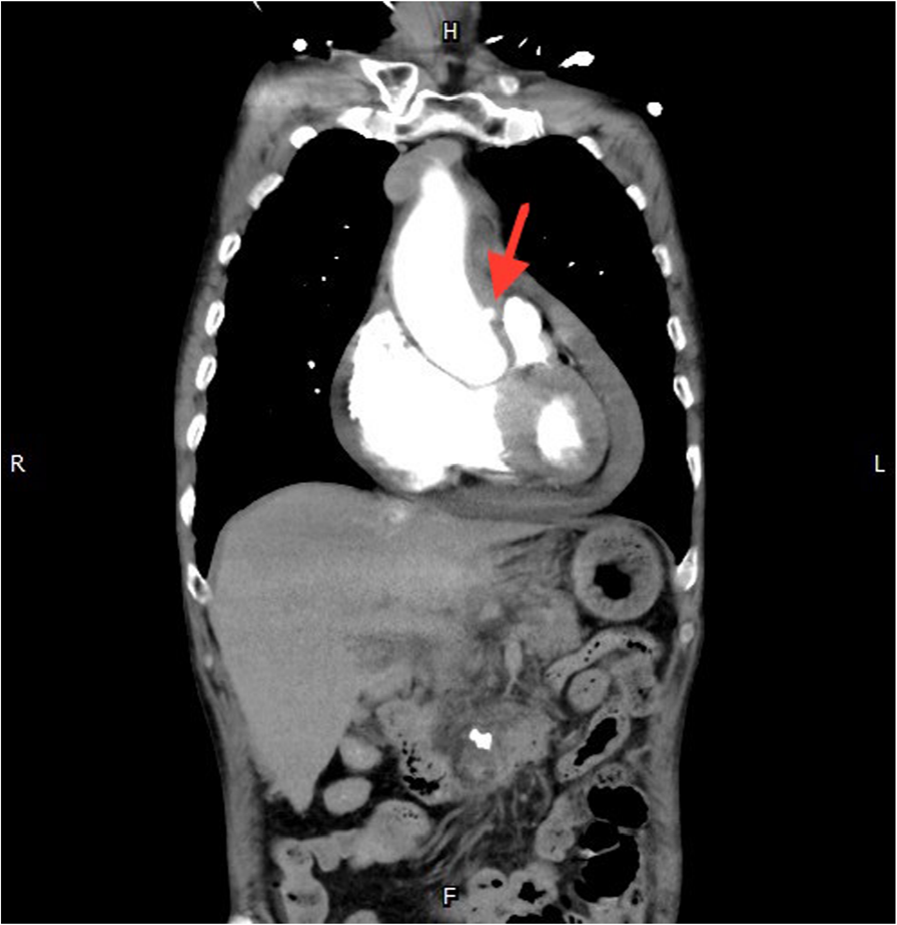
A protruding spot (PAU) in the ascending aorta, coronal view (red arrow)

**Fig. 2:**
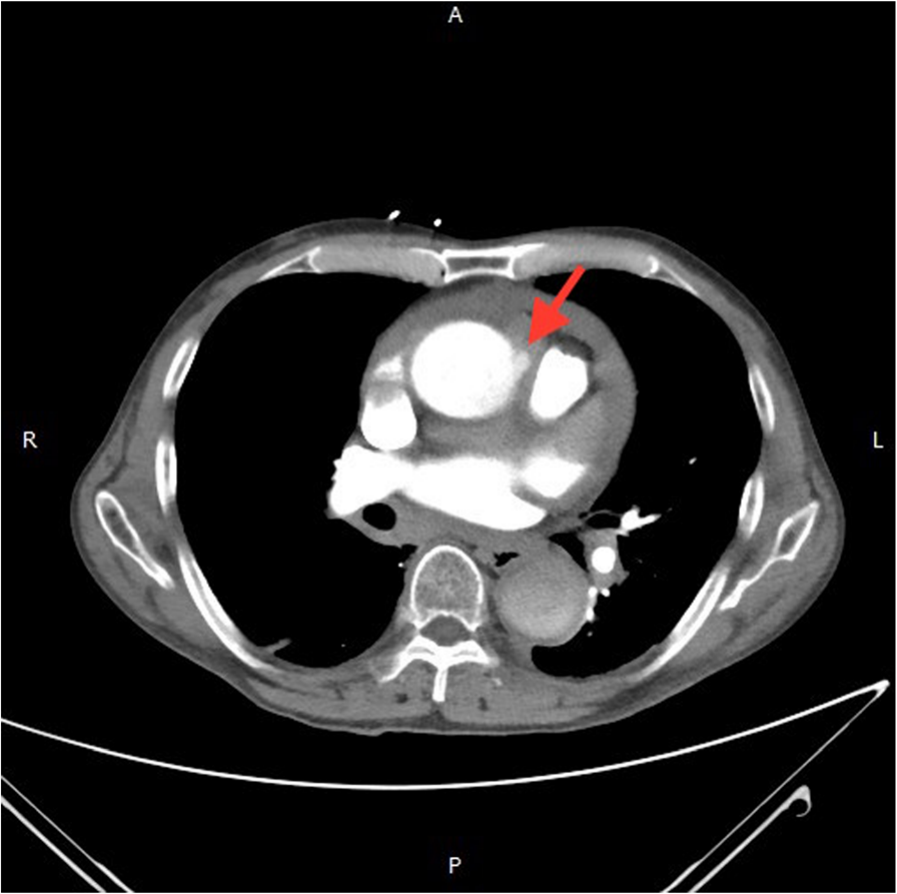
A protruding spot (PAU) in the ascending aorta, axial view (red arrow)

A 76-year-old man with a history of essential hypertension, who had not been taking his medications before this admission, presented to the emergency department (ED) because of a sudden chest pain at rest. His vital signs in the ED were a blood pressure of 82/60 mmHg, heart rate of 158 beats per min, respiratory rate of 22 breaths per min, and a body temperature of 37.2 °C. Electrocardiogram did not show an ST segment elevation, and cardiac enzymes were within normal limits. No widening mediastinum was found on chest radiography, but echocardiography revealed a large pericardial effusion with impending cardiac tamponade. Ruptured PAU of the ascending aorta with hemopericardium was diagnosed with chest CT scan (Figs. 1 and 2). A ruptured ulcer plaque through the intima to the adventitia without flap dissection in the ascending aorta was confirmed. An emergent sternotomy and ascending aorta reconstruction were performed. The postoperative recovery was uneventful, and the patient was discharged 18 days after the operation.

## Discussion

PAU tends to occur in elderly men with hypertension, tobacco use, and coronary artery disease [5]. This lesion may precipitate intramural hemorrhage, aortic aneurysm, pseudoaneurysm, or even aortic dissection. PAU usually involves the descending aorta, and although rare in the ascending aorta, it is commonly lethal [6] when it ruptures. Coady et al. [6] demonstrated that the rate of aortic rupture is markedly higher for intramural hematoma (35 %) and PAU (42 %) than for aortic dissection (type A 7.5 %, type B 4.1 %). PAU is frequently asymptomatic and is almost always incidentally diagnosed. The initial clinical appearance of PAU is similar to that of a classic aortic dissection. Differentiating PAU from the other AAS entities as well as acute pulmonary embolism and acute myocardial infarction is important. We can differentiate between all of these diseases in the ED based on patient history, physical examination, electrocardiography, cardiac biomarkers, D-dimers, transthoracic echocardiography, transesophageal echocardiography, chest radiography, CT scan, and magnetic resonance imaging. As CT scan provides a quick and high quality two- and three-dimensional image reconstruction, it is the best method for diagnosing PAU, which appears as a focal, contrast-filled outpouching with jagged margins that extends beyond the expected aortic wall boundaries, usually associated with severe underlying atheromatous disease [5]. Although PAU has a high risk of aortic rupture, some authors believe that immediate surgical treatment is not always required because the disease may have a benign course. In the study by Harris et al. [7], few patients developed aortic dissection or aortic rupture during follow-up. Besides, these authors emphasized that compared with the risk of acute aortic rupture, most patients with PAU were at a higher risk from surgical intervention because of their advanced age and poor general health. Moreover, surgical repair of the descending thoracic aorta is frequently associated with complications such as respiratory disease, renal insufficiency, or spinal ischemia. Based on these reasons, patients with PAU involving the descending aorta can initially be treated conservatively with aggressive medical therapy and close observation, similar to a descending aortic dissection [8]. Surgery becomes necessary when there are signs of expansion of intramural hematoma (IMH), rupture into the pericardial or pleural cavity, intractable chest pain, and hemodynamic instability. Besides, patients with a PAU must be followed up, particularly during the first month after the onset of AAS [2]. However, for patients with PAU in the ascending aorta, even asymptomatic patients, surgical intervention is recommended as soon as possible because they are at a high risk of evolving into a classic type A aortic dissection, type A IMH, or acute ascending aorta rupture with cardiac tamponade. Owing to improvement of endovascular techniques and advancement of devices, thoracic endovascular aortic repair (TEVAR) has become the first-line approach for aortic diseases because of its high technical success rate (96.4 %–98.3 %) and low morbidity and mortality rates [9, 10]. TEVAR has been proved to yield excellent short-term and mid-term results in PAU of the descending aorta and has been the first line of management when intervention is indicated. However, TEVAR of PAU in the ascending aorta is rare as PAU is usually found incidentally in asymptomatic patients and is complicated with aortic dissection, IMH, or acute aortic rupture in AAS patients. A conventional open repair is still the first consideration in most of these cases. Endovascular therapy of the ascending aorta is frequently used as a “rescue tool” outside of its intended indication. However, several studies have confirmed the safety and feasibility of endovascular repair of the ascending aorta for high-risk patients unsuitable for open repair [11, 12]. Most recently, Khoynezhad et al. [13] designed an FDA-approved physician-sponsored investigational device exemption study to investigate the outcome of endovascular repair of the ascending aorta. They demonstrated positive remodeling of the excluded aortic segments similar to surveillance studies involving the descending aorta. Although only six patients were enrolled in the study and only one of them was indicated due to an ascending PAU, we anticipate that endovascular repair may be feasible in patients with PAU in the ascending aorta in the future.

## Conclusion

PAU is frequently asymptomatic and is diagnosed rather incidentally. While a PAU in the ascending aorta is uncommon, it is commonly lethal when it ruptures. Generally, PAU in the descending aorta is conservatively treated with aggressive medical therapy and close observation similar to a descending aortic dissection. We recommend treating PAU in the ascending aorta with an aggressive surgical intervention. A traditional operative procedure involves a sternotomy and ascending aorta reconstruction. However, with the advances in endovascular techniques and devices, endovascular repair of PAU in the descending aorta has been the first line of management when intervention is indicated. Although endovascular repair of PAU in the ascending aorta is employed for high-risk patients who are currently unsuitable for open repair, we anticipate that endovascular repair may be feasible in patients with PAU in the ascending aorta in the future.

## Abbreviations

AAS, acute aortic syndrome; CXR, chest roentgenogram; ED, emergent department; IMH, intramural hematoma; PAU, penetrating atherosclerotic ulcer; TEVAR, thoracic endovascular aortic repair
